# Myofibroblast phagocytic cutaneous mucinosis: phagocytosis of mucinous substances by myofibroblasts in a distinctive cutaneous mucinosis: A case report

**DOI:** 10.1097/MD.0000000000020867

**Published:** 2020-07-17

**Authors:** Takeo Nakaya, Koji Kamiya, Michio Nakaya, Kentaro Tsuji, Toshiro Niki, Mamitaro Ohtsuki, Akira Tanaka

**Affiliations:** a Department of Pathology; b Department of Dermatology, Jichi Medical University, Tochigi; c Department of Pharmacology and Toxicology, Graduate School of Pharmaceutical Sciences, Kyushu University, Fukuoka, Japan.

**Keywords:** α-smooth muscle actin-positive myofibroblasts, cutaneous mucinosis, myofibroblast phagocytic cutaneous mucinosis, myofibroblasts, phagocytosis

## Abstract

**Rationale:**

Phagocytosis is an important physiological process for eliminating unnecessary substances or dead cells after tissue damage, such as inflammation or infarction. Phagocytosis was previously considered to be mainly performed by professional phagocytotic cells, such as macrophages. In contrast, we previously demonstrated that the phagocytosis of dead cells and unnecessary substances by myofibroblasts is as important as that by professional phagocytotic cells in myocardial infarction. Based on our discovery, we speculated that phagocytosis by myofibroblasts may be a more common pathological phenomenon also in other diseases than previously believed.

**Patient concerns:**

A 44-year-old male patient with atopic dermatitis developed a cutaneous reddish nodule with an underlying induration on his thigh.

**Interventions:**

The cutaneous lesion was surgically removed.

**Diagnoses:**

Histopathological examination demonstrated that the cutaneous lesion had solid infiltration by inflammatory cells, namely, plasma cells, histiocytes, and lymphocytes, in the dermis. The cutaneous lesion included mucinosis in the dermis. Inside the mucinosis, we detected cells with clear areas of mucinous substances. Some of the cells were α-smooth muscle actin-positive myofibroblasts. Electron microscopic images demonstrated that there were collagen bands in the cells with mucinous engulfment. Based on these pieces of evidence, we conclude that these mucinous phagocytotic cells were myofibroblasts, not professional phagocytotic cells, such as macrophages.

**Outcomes:**

There was no recurrence of the lesion.

**Lessons:**

The clinical appearance of this case resembled that of previously reported solitary cutaneous focal mucinoses. However, our case had distinctive characteristics, such as the phagocytosis of mucinous substances by myofibroblasts, multiple mucinous lesions in a single eruption, and the presence of inflammatory cells, which have not been previously reported. For this distinct cutaneous lesion, a clear dermatological and pathological name has yet to be determined. We propose “myofibroblast phagocytic cutaneous mucinosis” as a candidate name. In addition, our discoveries suggest that phagocytosis by myofibroblasts is not rare but rather is a common pathological phenomenon that has been undetected or unrecognized.

## 1 Introduction

Phagocytosis is an important physiological process for eliminating unnecessary substances or dead cells and is important for tissue remodeling after tissue damage, such as inflammation or infarction.^[1–3]^ Phagocytosis was previously believed to be mainly performed by professional phagocytotic cells, such as macrophages.^[4,5]^

However, it was observed that a time lag exists before professional phagocytotic cells are recruited to damaged tissues where unnecessary substances should be eliminated.^[6]^ Thus, some cells likely eliminate unnecessary substances before the recruitment of professional phagocytotic cells.

We previously demonstrated that the cardiac myofibroblast engulfment of dead cells facilitates recovery after myocardial infarction.^[6]^ Namely, the phagocytosis of dead cells and unnecessary substances by myofibroblasts is as important as that by professional phagocytotic cells in myocardial infarction. Myofibroblasts are not present in normal conditions but appear in damaged tissues after differentiating from several cell types in response to inflammation.^[7–9]^ Based on our discovery of myofibroblast phagocytosis in myocardial infarction, we speculated that myofibroblast phagocytosis is actually a common pathological phenomenon in other diseases than those that have been detected or recognized.

We encountered an interesting case of phagocytosis of mucinous substances in the skin. Based on our previous discovery about phagocytotic myofibroblasts in myocardial infarction, we predicted that myofibroblasts, along with professional phagocytotic cells, might take part in the phagocytosis of mucinous substances. As we expected, we found the phagocytosis of mucinous substances by α-smooth muscle actin (αSMA)-positive myofibroblasts in cutaneous inflammatory mucinosis. This case demonstrates that phagocytosis by myofibroblasts may be a common pathological phenomenon in other diseases that has been undetected or undiscovered.

## 2 Case presentation

### 2.1 Clinical history

**Figure 1:**
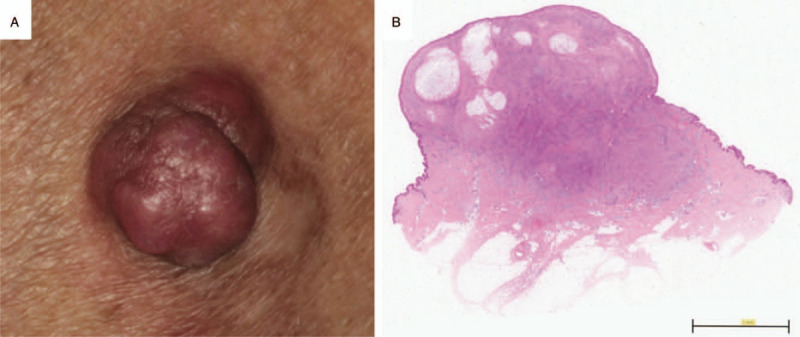
Clinical appearance and hematoxylin and eosin (H&E) staining of the histopathological microscopic view of the lesion. (A) The cutaneous reddish nodule on the right thigh. (B) The inflammatory and mucinous lesions in the skin of the right thigh (H&E staining, scale bar, 5 mm).

A 44-year-old male patient with atopic dermatitis developed a cutaneous reddish nodule with an underlying induration on his right thigh (Fig. 1A). The cutaneous lesion was recognized about 1 year before the surgical removal. The cutaneous lesion was surgically removed, and the resected specimen was pathologically examined as follows. The patient has provided informed consent for publication of the case.

The patient has no recurrence of this cutaneous lesion for about 2 years after the surgery.

### 2.2 Pathological findings

The surgical specimen was 50 × 23 mm of skin. The specimen had an 18 × 14-mm elevated lesion that included small mucinous cystic lesions.

The cutaneous lesion had the solid infiltration of inflammatory cells, namely, plasma cells, histiocytes, and lymphocytes, in the dermis (Fig. 1B). Most of the inflammatory cells consisted of CD138-positive plasma cells, which did not show light-chain restriction. This observation confirmed that the lesion was not a tumor but was inflammatory in nature.

S100-positive cells and CD68-positive cells were scattered in the fibrous tissue and in the areas of inflammatory cell infiltration. These cells were CD1a-negative, which demonstrated that these cells were not proliferating Langerhans cells.

The cutaneous lesion included mucinosis in the dermis (Fig. 1B). We speculated that the inflammatory cell infiltration induced the mucinosis.

**Figure 2:**
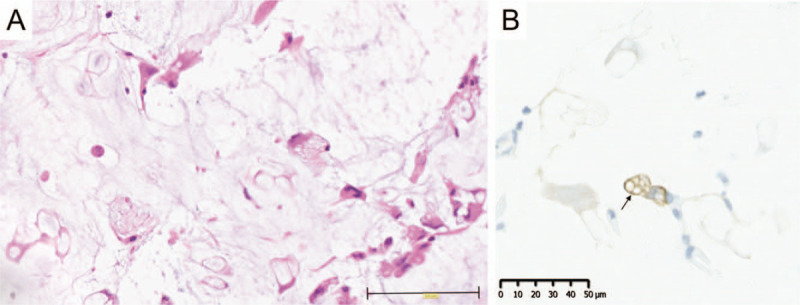
Phagocytosis of mucinous substances by αSMA-positive myofibroblasts during cutaneous inflammatory mucinosis. (A) Histopathology showing the mucinosis inside the lesion (H&E staining, scale bar, 100 μm). Phagocytosis of mucinous substances was observed. (B) Some of the cells that performed the phagocytosis of mucinous substances were αSMA-positive myofibroblasts (scale bar, 50 μm). The arrowhead shows strongly αSMA-positive cells.

Inside the mucinosis, we detected cells with clear areas of mucinous substances (Fig. 2A). First, we speculated that these cells were phagocytotic macrophages. Some cells inside the mucinosis were CD68-positive macrophages, whereas some cells were not. CD138-positive cells were not dominant in the mucinosis. The cells with clear areas of mucinous substances were cytokeratin AE1/AE3-negative, which showed that these cells were not epithelial cells, including metastatic carcinoma cells such as signet ring cell adenocarcinoma.

Recently, we discovered a novel function of myofibroblasts, wherein the engulfment of dead cells by cardiac myofibroblasts facilitated recovery after myocardial infarction.^[6]^ This finding suggested that the cells that engulfed the mucinous substances might include myofibroblasts that were phagocytizing mucinous substances during tissue remodeling. In fact, some of the cells were αSMA-positive myofibroblasts (Fig. 2B).

**Figure 3:**
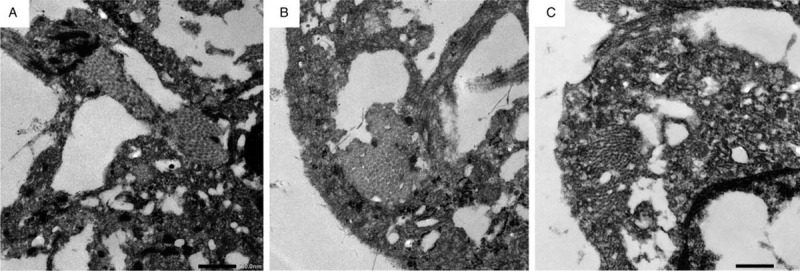
(A, B, C) Electron microscopic images demonstrated that there were collagen bands in the cells with mucinous engulfment.

Electron microscopic images also demonstrated that there were collagen bands in the cells with mucinous engulfment (Fig. 3A-C). The detection of collagen bands inside the cells by electron microscopy suggests 3 general possibilities: these cells were fibroblasts or myofibroblasts that generated collagen bands^[10]^; these cells had engulfed collagen fibers^[11]^; and collagen fibers from outside the cells were included in the microscopic section. We speculate that the cells with collagen fibers were myofibroblasts, although we cannot exclude the other 2 possibilities mentioned above.

Altogether, based on the detection of αSMA-positive phagocytotic cells by immunohistochemistry and collagen bands in phagocytotic cells by electron microscopy, we conclude that these phagocytotic cells were myofibroblasts, not professional phagocytotic cells, such as macrophages (Fig. 2A-B, Fig. 3A-C).

## 3 Discussion

In summary, we speculate that the pathological formation of this cutaneous lesion occurred in the following way. First, inflammatory cell infiltration occurred. Second, the accumulation of inflammatory cells, such as plasma cells, generated the subsequent mucinosis in the dermis. Third, the myofibroblasts were induced before the appearance of professional phagocytotic cells, and some of the myofibroblasts performed the phagocytosis of the mucinous substances to promote tissue remodeling by eliminating unnecessary mucinous substances.

In this case, we described the phagocytosis of mucinous substances by myofibroblasts, based on the engulfment of mucinous substances by αSMA-positive phagocytotic cells and the existence of collagen bands in the mucinous phagocytotic cells. Although we cannot exclude other possibilities, such as the engulfment of collagen fibers by phagocytotic cells,^[11]^ the collagen fibers seemed to be included in the cytoplasm of phagocytotic cells and not included inside the mucinous cysts, in the electron microscopic pictures (Fig. 3A-C). In combination with the fact that the phagocytotic cells were αSMA-positive, these observations strongly support the phagocytosis by myofibroblasts in this cutaneous lesion.

Myofibroblasts mainly originate from resident cardiac fibroblasts that perform the phagocytosis of dead cells before the induction of professional phagocytotic cells, such as macrophages, in myocardial infarction.^[6]^

Although the cutaneous lesion on the thigh was different from myofibroblasts in myocardial infarction, the histopathological formation and time course of this cutaneous case might resemble those of cardiac tissue damaged by myocardial infarction. Cutaneous tissue is rich in fibroblasts. Thus, activated myofibroblasts might be generated or transformed from residual fibroblasts before the recruitment of professional phagocytotic cells to a mucinous lesion. The inflammation caused by plasma cells and other inflammatory cells might facilitate the fibroblast-to-myofibroblast transition.^[12]^ Our observations in a cutaneous lesion and in myocardial infarction suggest that phagocytosis by myofibroblasts is not rare but rather is a common pathological phenomenon that has been undetected or unrecognized.^[6]^

The clinical appearance of this case resembled that of previously reported solitary cutaneous focal mucinoses (Fig. 1A).^[13–16]^ However, our case had distinctive characteristics from previously reported cutaneous focal mucinosis cases, such as the phagocytosis of mucinous substances by myofibroblasts, multiple mucinous lesions in a single eruption, and the presence of inflammatory cells (Fig. 1B), which have not been previously reported.

As for this distinct cutaneous lesion, a clear dermatological and pathological name has yet to be determined. We propose “myofibroblast phagocytic cutaneous mucinosis” as a candidate name. Intensive examination might reveal cases of this disease that would otherwise remain undetected.

This case gives us profound insights into the distinctive and essential role of myofibroblasts in the early phase of phagocytosis in pathophysiologies.

## Acknowledgments

The thank the support from the members of Department of Pathology and Dermatology, Jichi Medical University.

## Author contributions

**Conceptualization:** Takeo Nakaya, Michio Nakaya, Toshiro Niki, Mamitaro Ohtsuki, Akira Tanaka.

**Data curation:** Takeo Nakaya, Koji Kamiya, Michio Nakaya, Kentaro Tsuji, Toshiro Niki, Akira Tanaka.

**Formal analysis:** Takeo Nakaya, Koji Kamiya, Michio Nakaya, Kentaro Tsuji, Toshiro Niki, Akira Tanaka.

**Investigation:** Takeo Nakaya, Koji Kamiya, Michio Nakaya, Kentaro Tsuji, Toshiro Niki, Mamitaro Ohtsuki, Akira Tanaka.

**Methodology:** Takeo Nakaya, Koji Kamiya, Michio Nakaya, Kentaro Tsuji, Toshiro Niki, Mamitaro Ohtsuki, Akira Tanaka.

**Project administration:** Takeo Nakaya, Toshiro Niki, Mamitaro Ohtsuki, Akira Tanaka.

**Resources:** Takeo Nakaya, Koji Kamiya, Kentaro Tsuji, Toshiro Niki, Mamitaro Ohtsuki, Akira Tanaka.

**Software:** Takeo Nakaya.

**Supervision:** Takeo Nakaya, Michio Nakaya, Toshiro Niki, Mamitaro Ohtsuki, Akira Tanaka.

**Validation:** Takeo Nakaya, Koji Kamiya, Michio Nakaya, Toshiro Niki, Mamitaro Ohtsuki, Akira Tanaka.

**Visualization:** Takeo Nakaya, Koji Kamiya.

**Writing – original draft:** Takeo Nakaya.

**Writing – review & editing:** Takeo Nakaya, Koji Kamiya, Michio Nakaya, Toshiro Niki, Mamitaro Ohtsuki, Akira Tanaka.

## References

[1] Nagata S Apoptosis and clearance of apoptotic cells. Ann Rev Immunol 2018;36:489–517. 29400998 10.1146/annurev-immunol-042617-053010

[2] Nagata S Hanayama R Kawane K Autoimmunity and the clearance of dead cells. Cell 2010;140:619–30. 20211132 10.1016/j.cell.2010.02.014

[3] Nakaya M Tajima M Kosako H GRK6 deficiency in mice causes autoimmune disease due to impaired apoptotic cell clearance. Nat Commun 2013;4. 10.1038/ncomms2540 PMC3586722 23443560

[4] Frangogiannis NG Regulation of the inflammatory response in cardiac repair. Circ Res 2012;110:159–73. 22223212 10.1161/CIRCRESAHA.111.243162 PMC3690135

[5] Wan E Yeap XY Dehn S Enhanced efferocytosis of apoptotic cardiomyocytes through myeloid-epithelial-reproductive tyrosine kinase links acute inflammation resolution to cardiac repair after infarction. Circ Res 2013;113:1004–12. 23836795 10.1161/CIRCRESAHA.113.301198 PMC3840464

[6] Nakaya M Watari K Tajima M Cardiac myofibroblast engulfment of dead cells facilitates recovery after myocardial infarction. J Clin Invest 2017;127:383–401. 27918308 10.1172/JCI83822 PMC5199696

[7] Gabbiani G The myofibroblast in wound healing and fibrocontractive diseases. J Pathol 2003;200:500–3. 12845617 10.1002/path.1427

[8] Zeisberg M Kalluri R Cellular Mechanisms of Tissue Fibrosis. 1. Common and organ-specific mechanisms associated with tissue fibrosis. Am J Physiol-Cell Ph 2013;304:C216–25. 10.1152/ajpcell.00328.2012 PMC3566435 23255577

[9] Gabbiani G The biology of the myofibroblast. Kidney Int 1992;41:530–2. 1573823 10.1038/ki.1992.75

[10] Ghadially FN Lalonde JMA Yong NK Myofibroblasts and intracellular collagen in torn semilunar cartilages. J Submicr Cytol Path 1980;12:447–55.

[11] Pearson RW Studies on the pathogenesis of epidermolysis bullosa. J Invest Dermatol 1962;39:551–75. 13942265 10.1038/jid.1962.156

[12] Reeves SR Kolstad T Lien TY Fibroblast-myofibroblast transition is differentially regulated by bronchial epithelial cells from asthmatic children. Respir Res 2015;16:21. 25849331 10.1186/s12931-015-0185-7 PMC4333174

[13] Johnson WC Helwig EB Cutaneous focal mucinosis. A clinicopathological and histochemical study. Arch Dermatol 1966;93:13–20. 4221380

[14] Kuo KL Lee LY Kuo TT Solitary cutaneous focal mucinosis: a clinicopathological study of 11 cases of soft fibroma-like cutaneous mucinous lesions. J Dermatol 2017;44:335–8. 27450934 10.1111/1346-8138.13523

[15] Rongioletti F Rebora A Cutaneous mucinoses—microscopic criteria for diagnosis. Am J Dermatopath 2001;23:257–67. 11391115 10.1097/00000372-200106000-00022

[16] Wilk M Schmoeckel C Cutaneous focal mucinosis—a histopathological and immunohistochemical analysis of 11 cases. Journal of Cutaneous Pathology 1994;21:446–52. 7532654 10.1111/j.1600-0560.1994.tb00287.x

